# Genetic diversity of the cultivated *Salvia miltiorrhiza* populations revealed by four intergenic spacers

**DOI:** 10.1371/journal.pone.0266536

**Published:** 2022-04-06

**Authors:** Jie Feng, Fang Liao, Deying Kong, Ruihua Ren, Tao Sun, Wei Liu, Yanyan Yin, Haoyu Ma, Jiahao Tang, Guanrong Li

**Affiliations:** 1 College of Agronomy and Biotechnology, Southwest University, Chongqing, China; 2 Animal, Plant and Foodstuff Inspection Center, Tianjin Customs, Tianjin, China; 3 Technology Center, Chongqing Customs, Chongqing, China; National Agri-Food Biotechnology Institute (NABI) Mohali, INDIA

## Abstract

For better understanding the genetic diversity and phylogeny of the cultivated *Salvia miltiorrhiza* populations, four intergenic spacer sequences, *ETS*, *psbA-trnH*, *trnL-trnF*, and *ycf1-rps15* of the 40 populations collected from China were Polymerase Chain Reaction (PCR) amplified, analyzed both individually and in combination. Haplotype diversity analysis showed that the cultivated *S*. *miltiorrhiza* populations had a very rich genetic diversity and an excellent capacity to resist environmental pressure. The best-fit nucleotide substitution models for *ETS*, *psbA-trnH*, *trnL-trnF*, *ycf1-rps15*, and their combined sequences were HKY+I, T92, T92, T92+G, and T92+G, respectively; the nucleotide conversion frequency in the combined sequences was lower than the transversion, and the relatively high nucleotide substitution frequencies suggests its high genetic variability. Neutral tests showed that the spacer sequences of the populations conform with the neutral evolution model, and there has been no current expansion events occurred. Phylogeny analyses based on both the individual and the combined sequences showed that the 40 populations were clustered in two clades with a very similar topological structure. The discrimination rate of the combined sequence marker is significantly increased to 52.5% (21 populations) over the highest 35% (13 populations) by the single marker of *ETS*, though still inadequate but a big step forward. Further exploration of more DNA markers is needed. This study for the first time revealed the rich genetic diversity and phylogeny of the currently cultivated *S*. *miltiorrhiza* populations in China and provides novel alternative molecular markers for the genetic identification and resources evaluation of the cultivated *S*. *miltiorrhiza* populations.

## Introduction

*Salvia miltiorrhiza Bunge* is a perennial medicinal plant of *Salvia* in the Labiatae family [1]. Its rhizomes have been traditionally and widely used as medicine in China, Japan, the United States and Europe to treat cardiovascular disorders, including atherosclerosis, high blood pressure, hyperlipidemia and stroke [2]. In the last decade, it has been also proved that *S*. *miltiorrhiza* has many other pharmacological effects, such as antioxidant [3], neuroprotective [4], anti-fibrosis [5], anti-inflammatory and antibacterial [6], and anti-tumor [7]; and its extensive application has been contributing greatly to patients care and human health. Although native to China, *S*. *miltiorrhiza* is also widely cultivated in other countries such as South Korea, Vietnam and Australia [8]. The pharmaceutically effective ingredients of *S*. *miltiorrhiza* mainly include fat-soluble tanshinones and water-soluble phenolic acids [9].

Genetic diversity, the genetic variability present within species that changes with time and space, is the product of recombination of genetic material by sexual reproduction, and by mutation of genes, genetic drift, and gene flow. It gives different physical attributes to the individual and capacity to adapt to stress, diseases and unfavorable environmental conditions. Low genetic diversity is an important factor affecting species survival, leading to endangerment or even to distinction. The genetic diversity at various levels provides the basis for the collection, preservation, utilization and evaluation. To ensure and maintain good levels of genetic diversity in crop populations is crucial. The genetic diversity at various levels provides the basis for the collection, preservation, utilization and evaluation of *S*. *miltiorrhiza*.

The external properties of *S*. *miltiorrhiza* cultivated in different producing areas are very similar and difficult to identify, and the yields and medicinal ingredient contents of different germplasm are also different, which affects the stability of the quality of *S*. *miltiorrhiza* medicinal materials and brings multiple difficulties to the standardized production of *S*. *miltiorrhiza* [10]. At present, traditional methods based on sources, morphological traits, physical and chemical and biological properties have been mainly used to identify the provenance of *S*. *miltiorrhiza*. They have played important roles in the identification and quality evaluation of Chinese medicinal materials, but also have their own shortcomings. Trait identification is difficult to identify materials with similar morphological traits, the microscopic identification requires the relevant professional knowledge, and the physical and chemical properties are easily affected by many environmental factors. In view of the limitations of traditional identification, new methods as supplements have been emerging to ensure more accurate and reliable identification. In recent years, molecular marker technology that reveals polymorphism at the DNA level has become a powerful tool for studying plant genetic diversity [11]. Both the chloroplast and nuclear gene markers are excellent for application for these purposes.

The nuclear ribosomal DNAs (nrDNAs) in higher plants are highly tandem repeating sequences consisted of the external transcribed spacer (*ETS*), the non-transcribed spacer (*NTS*), 18SrDNA, internal transcription spacer 1 (*ITS1*), 5.8SrDNA, internal transcription spacer 2 (*ITS2*), and 28SrDNA. The non-coding regions of nrDNA are hypervariable, due to the much less selective pressure they subjected. Sequence differences manifested in these regions have potentials for application in systematics, hybridization and population evolution researches among closely related groups [12]. Seven markers had been selected to barcode a wide range of medicinal plant species and their relatives, and found that *ITS2* reached the highest discriminating rate of 92.7% at the species level among the 7 fragments [13]. The relatively less repetitive *ETS* evolves rapidly and has a high degree of polymorphism and variability, and is extensively used in the study of genetic variation, classification and phylogeny. For example, the phylogenetic study on the origin and spread of millet based on *ETS* detected both intra- and inter-species polymorphisms [14]; and analysis of the *ETS* of sycamore showed that all the Scandinavian populations composed of well-defined subgroups of the species’ genes and had a spontaneous origin closest with other spontaneous populations from neighboring areas of the Nordic continent [15]. The genetic diversity of 39 Chinese cultivated and wild pepper populations from 8 provinces has been assessed by two nrDNA non-coding markers (*ETS* and *ITS2*) and found that despite its long-term cultivation, the genetic variation was relatively high existing mainly within each province rather than between provinces [16].

The chloroplasts of plants have higher evolving rates than mitochondria; its uniparental inheritance enables the researchers to target DNA markers in the genome [17]. Initially, functional genes for rubisco large subunit (*rbcL*), maturase K (*matK*), NAD(P)H dehydrogenase F (*ndhF*), the β-subunit of ATP synthase (*atpB*), the RNA polymerase B and C subunit (*rpoB* and *rpoC*) genes, and intergenic spacers between tRNA^Leu^ and tRNA^Phe^ genes (*trnL-trnF*), between D1 protein of PSII and tRNA^His^ genes (*psbA-trnH*), and between CP47 protein and small subunit I genes of PSII (*psbK-psb*I) in the chloroplast genome were used individually for plant species identification. Later, it was proposed to use different combinations of these fragments. For example, the relatively conserved *psbA* [18] has been studied as markers in lake and marine ecosystems [19]; the second longest hypothetical chloroplast open reading frame1(*ycf1*) in the chloroplast genome [20] which has been proved to be essential for plant survival [21] has only recently begun to be explored in phylogenetic studies; the chloroplast *rps15* gene encoding a ribosomal protein S15 (rps15), mediating the interaction between the subunits and interacting with mRNA and tRNA [22] plays certain role in the production of ribosomes [23]. It has been also used for phylogenetic studies [13].

Current researches on plant genetic geography have been under transition from individual sequences in the past to the joint analysis of the sequences from the chloroplast genome or nuclear ribosomal genes. For example, the *trnH-psbA* spacer and partial coding region of *rbcL* gene has been combined as two global terrestrial plant molecular genetic markers and provided the necessary versatility and species differentiation [24]. A higher species resolution has been obtained with the combination of *matK* + *atpF-atpH* + *psbK-psbI* [25]; the monophyly and placement of *Lepechina* in the Lamiaceae plants have been tested based on separate and combined datasets of cpDNA (*ycf1*, *ycf1-rps15*, and *trnL-trnF*) and the combined nrDNA (*ITS* and *ETS*) datasets [26]. The phylogeny and biogeographic history of Lamiaceae plants based on a combined sequence of 1144bp containing 519 mutation sites and 339 parsimony information sites from five plastid sequences (*rbcL*, *rps16*, *rpl32-trnH*, *psbA-trnH* and *trnL-trnF*) and two nuclear sequences (*ITS* and *ETS*) has been explored to interpret the disjunct distribution of Northern Hemisphere herbaceous plants [27]. The phylogeny of East Asian sage of the most comprehensive geographic location, taxonomy, and genetic sampling data has been reconstructed by two separate matrixes, one combined the two nrDNA (*ITS*, *ETS*), and the other combined the four cpDNA (*psbA-trnH*, *ycf1-rps15*, *trnL-trnF*, and *rbcL*) [28].

The chloroplast genome of *S*. *miltiorrhiza* has been sequenced and its detailed structure provides an excellent basis for systematic analyses [29]. Its more variable non-coding regions could be readily used for intra- and inter-specific phylogenetic studies.

As one of China’s traditional bulk medicinal materials, *S*. *miltiorrhiza* has been playing increasingly important roles in clinical and health-care utilization. However, the random introduction of unidentified source varieties in production and nonstandard management have resulted in a chaotic variety sources and unstable quality; and studies concerning the genetic diversity and molecular identification of the germplasm resources have been also inadequate. So far as we known, no reports on the genetic diversity of the cultivated *S*. *miltiorrhiza* populations have been seen. In this study, the nuclear *ETS* and three chloroplast intergenic spacers of the 40 cultivated *S*. *miltiorrhiza* populations in China were for the first time analyzed in order to identify the SNP fingerprints, understand the genetic diversity and phylogeny both individually and in combination. A basis for the protection, introduction, domestication, novel variety selection, and breeding of the cultivated *S*. *miltiorrhiza* germplasm resources, was provided.

## Materials and methods

### Sampling

Seeds of 40 cultivated *S*. *miltiorrhiza* populations representing more than 30 regions of China were collected from three major seed industries (Table 1). Uniform seeds preliminarily selected were used for sowing in Southwest University Agricultural Station, Chongqing; and leaves of morphologically representative single plants of each population were used for extraction of genomic DNAs.

**Table 1:** Cultivated *S*. *miltiorrhiza* populations used in this study.

No.	Population/Voucher	Production Region	Source	No.	Population/Voucher	Production Region	Source
01	V-HBAG-V-2	Anguo, Hebei Hebei	Fenghong Seed Industry	21	B-SCZJ-V-2	Zhongjiang, Sichuan	Hengda Seed Industry
02	V-CQ-V-2	Chongqing	Hengda Seed Industry	22	B-SD-V-1	Shandong	Tongda Seed Industry
03	V-JXJA-V-2	Ji-an, Jiangxi Jiangxi	Hengda Seed Industry	23	R-HBAG-V-2	Anguo, Hebei	Fenghong Seed industry
04	V-JLCC-V-2	Changchun, Jilin	Hengda Seed Industry	24	R-HNFC-V-2	Fangcheng, Henan	Hengda Seed Industry
05	V-JSSY-V-2	Shuyang, Jiangsu	Hengda Seed Industry	25	R-SDJX-V-2	Juxian, Shandong	Hengda Seed Industry
06	V-GZ-V-1	Guizhou	Tongda Seed Industry	26	R-GX-V-2	Guangxi	Hengda Seed Industry
07	V-GD-V-1	Guangdong	Tongda Seed Industry	27	R-NM-V-2	Nemeng	Hengda Seed Industry
08	V-GD-V-2	Guangdong	Hengda Seed Industry	28	R-HNCS-V-2	Changsha, Hunan Hunan	Hengda Seed Industry
09	V-GSLX-V-2	Longxi, Gansu	Fenghong Seed industry	29	R-GSJQ-V-2	Jiuquan, Gansu	Hengda Seed Industry
10	V-BJ-V-2	Beijing	Fenghong Seed industry	30	W-SCHY-W-1	Hongyuan, Sichuan	Self-collected
11	V-YNLJ-V-2	Lijiang, Yunnan	Hengda Seed Industry	31	W-SXXA-bV-2	Xi’an, Shaanxi	Hengda Seed Industry
12	V-GZZY-V-2	Zunyi, Guizhou	Hengda Seed Industry	32	W-LNSY-V-2	Shenyang, Liaoning	Hengda Seed Industry
13	V-SC-V-1	Sichuan	Tongda Seed Industry	33	W-FJLY-V-2	Luoyuan, Fujian	Hengda Seed Industry
14	V-SD-V-1	Shandong	Tongda Seed Industry	34	W-GZ-V-1	Guizhou	Tongda Seed Industry
15	V-JS-V-1	Jiangsu	Tongda Seed Industry	35	W-SD-V-1	Shandong	Tongda Seed Industry
16	V-HNYZ-bV-2	Yongzhou, Hunan	Hengda Seed Industry	36	W-JS-V-1	Jiangsu	Tongda Seed Industry
17	B-SC-V-1	Sichuan	Tongda Seed Industry	37	W-SC-V-1	Sichuan	Tongda Seed Industry
18	B-AHQJ-V-2	Quanjiao, Anhui	Hengda Seed Industry	38	W-GD-V-1	Guangdong	Tongda Seed Industry
19	B-GD-V-1	Guangdong	Tongda Seed Industry	39	W-HBJM-V-2	Jingmen Hubei	Hengda Seed Industry
20	B-JS-V-1	Jiangsu	Tongda Seed Industry	40	W-YNLJ-V-2	Lijiang, Yunnan	Hengda Seed Industry

### Methods

#### Genomic DNA extraction

The total genomic DNA of *S*. *miltiorrhiza* was extracted by the CTAB method [30], the purity was confirmed by 1% agarose gel electrophoresis, and was stored at -20°C.

#### Primers and PCR amplification

Well-documented 4 primer pairs respectively for the 4 intergenic spacers (Table 2) were adopted. The spacers were PCR amplified in a 25μl reaction system consisted of 2×SanTaq PCR Mix (Sangon, Shanghai, China) 11μl, primers (10μmol/L) each 1μl, DNA template 1μl, ddH_2_O 11μl, with the program of pre-denaturation at 94°C for 5min, denaturation at 94°C for 30s, annealing for 30s, extension at 72°C for 30s, 35 cycles, and a final extension at 72°C for 10min. Products were electrophoresed by agarose gel, purified and then bidirectionally sequenced by dideoxy chain termination (Sangon, Chengdu, China).

**Table 2:** Primers used in this study.

Locus	Primer code	Sequences(5’→3’)	Ta (°C)	Length (nt)	Reference
*ETS*	ETS-bdf1 (F)	ATAGAGCGCGTGAGTGGTG	55	19	[31]
	18S-IGS (R)	GACAAGCATATGACTGGATCAA		22	[32]
*psbA-trnH*	psbAF	GTTATGCATGAACGTAATGCTC	55	22	[33]
	trnHR	CGCGCATGGTGGATTCACAAATC		23	[33]
*trnL-trnF*	trn-c (F)	CGAAATCGGTAGACGCTACG	57	20	[34]
	trn-f (R)	ATTTGAACTGGTGACACGAG		20	[34]
*ycf1–rps15*	ycf15711f	CTTGTATGRATCGTTATTGKTTTG	53	24	[26]
	ycf1rps15r	CAATTYCAAATGTGAAGTAAGTCTCC		26	[26]

Notes: F(f) and R(r): Forward and reverse primers respectively; Ta: Annealing temperature; nt: Nucleotides.

#### Data processing

The manually checked and confirmed sequence data were subject to BLAST analysis and a corresponding reference sequence with the highest identity percentage for each locus was determined and used for manual finishing and multiple alignment with BioEdit 7.09 and Vector NTI Advance 11.5.3, respectively. The best nucleotide substitution models were identified via the Find Best DNA/Protein Models in MEGA 7.0 [35], and the displayed substitution frequencies were used to make histograms with Excel. MEGA 7.0 was used for genetic evolution analysis and phylogenetic tree construction; unrooted phylogenetic trees with a cut-off value of 50% for consensus were constructed with MEGA 7.0 based on Neighbor-Joining (NJ) with a bootstrap value of 1000. DnaSP v5 was used for analyses of polymorphic sites, genetic diversity index, and neutral detection [36].

## Results

### General features of the intergenic spacers

The four intergenic spacer sequences of *ETS*, *psbA-trnH*, *trnL-trnF* and *ycf1-rps15* of the 40 cultivated *S*. *miltiorrhiza* populations were assorted and submitted to GenBank, and the corresponding accession numbers were obtained (S1 Table). Comparison analyses with the highest identity reference sequences, MG824361.1, KJ025055.1, KC414292.1 and MG824106.1 respectively for *ETS*, *psbA-trnH*, *trnL-trnF* and *ycf1-rps15*, showed that the *ETS* sequences of the *S*. *miltiorrhiza* populations was 421–433 bp in length, with a GC content of 56.8~62.8%, a variable rate as high as 54.8%, and a total of 241 SNP variable sites consisting of 205 parsimony informative sites, 36 singleton variable sites; the *psbA-trnH* was 335~348bp with a GC content of 22.4~26.2%, a variable rate of 24.7%, and a total of 90 SNP variable sites consisting of 71 parsimony informative site and 19 singleton variable sites; the *trnL-trnF* was 298~306bp with a GC content of 35.0~36.9%, a variable rate of 7.7%, and a total of 25 SNP variable sites consisting of 24 parsimony informative sites and 1 singleton information site; the *ycf1-rps15* was 421-433bp with a GC content of 25.9–30.5%, a variable rate of 23.1%, and a total of 105 SNP variable sites consisting of 88 parsimony informative sites and 17 singleton variable sites. The combined sequence length (in the order of *ETS*, *psbA-trnH*, *trnL-trnF*, and *ycf1-rps15*), was 1488-1517bp, with a GC content of 35.9–38.8%, a variable rate of 29.4%, and a total of 466 SNP variable sites consisting of 392 parsimony informative sites and 74 singlet variable sites (Table 3).

**Table 3:** General features of the intergenic spacers of the cultivated *S*. *miltiorrhiza* populations.

Intergenic spacer	*ETS*	*psbA-trnH*	*trnL-trnF*	*ycf1–rps15*	Combined seq.
Length range (bp)	421~433	335~348	298~306	431~434	1488–1517
Matrix length	440	364	325	455	1584
Average GC (%)	56.8~62.8	22.4~26.2	35.0~36.9	25.9~30.5	35.9–38.8
Sites with alignment gaps or missing data	36	46	43	55	179
Variable sites (%)	241 (54.8)	90 (24.7)	25 (7.7)	105 (23.1)	466 (29.4)
Singleton variable sites	36	19	1	17	74
Parsimony informative sites (%)	205 (46.6)	71 (19.5)	24 (7.4)	88 (19.3)	392 (24.7)
Best fit model	HKY+I	T92	T92	T92+G	T92+G
Relative identification (%)	14 (35.0)	8 (20.0)	4 (10.0)	11 (27.5)	21 (52.5)

Based on the matrix length of *ETS*, *psbA-trnH*, *trnL-trnF*, *ycf1-rps15*, or the combined sequence (Table 3), the parsimony informative sites accounted for 46.6, 19.5, 7.4, 19.3, and 24.7% respectively, indicating that the *ETS* of the cultivated *S*. *miltiorrhiza* populations had the highest in parsimony information site rate, the combined sequence, second; the chloroplast genome *psbA-trnH* and *ycf1-rps15*, lower and comparable, and *trnL-trnF* was the least.

In the studying of the processes of gene evolution, researchers have proposed different nucleotide substitution models to demonstrate the DNA substitution process. By MAGA 7.0 using the maximum likelihood method, the best nucleotide substitution models for the four DNA molecular markers under this study, *ETS*, *psbA-trnH*, *trnL-trnF*, *ycf1-rps15*, and the combined sequence were revealed to be HKY+I, T92, T92 and T92+G, and T92+G, respectively (Table 3).

The specific SNP fingerprints based on the *ETS* can discriminate 14 *S*. *miltiorrhiza* populations (V-JXJA-V-2, V-GD-V-1, V-GSLX-V-2, V-SC-V-1, V -SD-V-1, V-JS-V-1, B-GD-V-1, B-SD-V-1, W-SCHY-W-1, W-GZ-V-1, W-SD-V-1, W-GD-V-1, W-HBJM-V-2 and W-YNLJ-V-2) with a relative identification rate of 35%, the specific SNP fingerprints of the *psbA-trnH* can distinguish 8 *S*. *miltiorrhiza* populations (V- GD-V-2, V-BJ-V-2, V-HNYZ-bV-2, B-JS-V-1, B-SD-V-1, W-JS-V-1, W-SC- V-1 and W-GD-V-1) with a relative identification of 20%, the specific SNP fingerprints of *trnL-trnF* can distinguish 4 *S*. *miltiorrhiza* populations (V-HBAG-V-2, V-SC-V-1, R-GSJQ -V-2 and W-SC-V-1) with a relative identification of 10%, the specific SNP fingerprints of *ycf1-rps15* can distinguish 11 *S*. *miltiorrhiza* populations (V-SC-V-1, V-SD-V-1, V- JS-V-1, B-GD-V-1, B-JS-V-1, B-SD-V-1, W-GZ-V-1, W-JS-V-1, W-SC-V-1, W-GD-V-1 and W-YNLJ-V-2) with a relative identification of 27.5%. Specific SNP fingerprints based on the composite DNA marker could discriminate 21 *S*. *miltiorrhiza* populations (V-HBAG-V-2, V-JXJA-V-2, V-GD-V-1, V-GSLX-V-2, V-SC-V-1, V-SD-V-1, V-JS-V-1, V- GD-V-2, V-BJ-V-2, V-HNYZ-bV-2, B-GD-V-1, B-SD-V-1, B-JS-V-1, R-GSJQ-V-2, W-SCHY-W-1, W-SD-V-1, W-GD-V-1, W-HBJM-V-2, W-YNLJ-V-2, W-JS-V-1, W-SC-V-1) with a discrimination rate of 52.5%.

The results demonstrated that highest relative identification rate was by the combined sequence marker. For single markers, the relative identification rate by *ETS* sequence was the highest (Table 3).

### Nucleotide variation frequency analysis of the intergenic spacers

**Fig 1:**
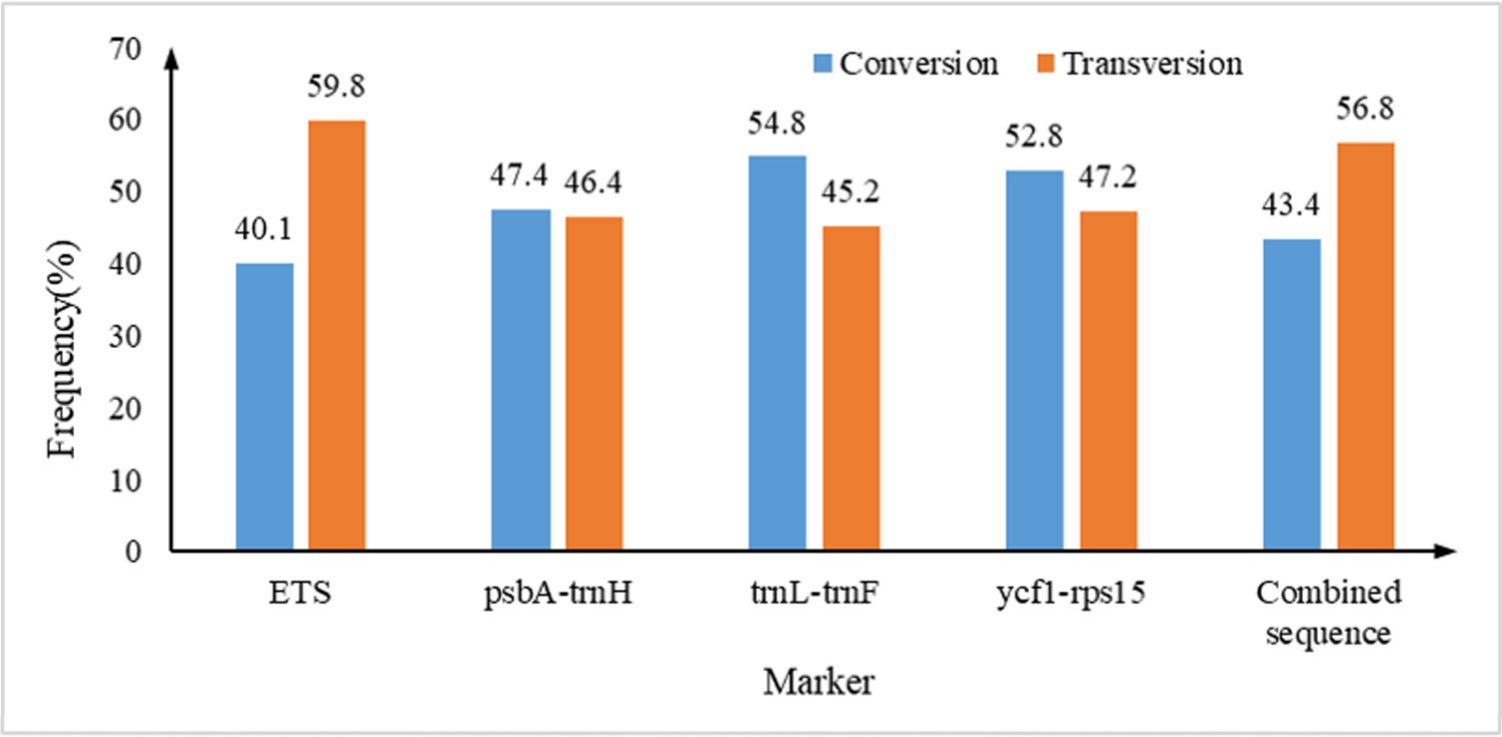
Nucleotide substitution frequencies of the individual and combined intergenic spacers of the cultivated *S*. *miltiorrhiza* populations.

Statistics on the nucleotide substitution frequencies (Fig 1) showed that for the nuclear ribosomal *ETS* sequences, the conversion frequency (40.1%) is lower than the transversion (59.9%), while for the other three intergenic spacers of the chloroplast genome, conversion frequencies were higher than those of transversion. The conversion and transversion frequencies of the combined sequences were 43.4% and 56.8% respectively, in good accordance with the those of the three non-coding regions of the chloroplast genome.

### Genetic diversity analysis of the intergenic spacers

Dna SP 5.10 was used to analyze the ploidy polymorphism and neutrality detection, and the haplotype, haplotype diversity (Hd), nucleotide diversity (π), variance of haplotype diversity (Vh) and standard deviation of haplotype diversity (Sh) of the four intergenic regions and the combined sequence. The results of neutral analysis showed that the Fu and Li’s D* and F* test statistics for *ETS* were 0.22815 and -0.37550 respectively, and the Tajima’s D was -1.27644, not significant at the level of P> 0.10, indicating its conformity with the neutral evolution model; the Fu and Li’s D* and F* test statistics for the *psbA-trnH* were 0.19802 and 0.53273 respectively, and the Tajima’s D was 0.90040, not significant at the level of P> 0.10, conforming also with the neutral evolution model; the Fu and Li’s D* test statistic for the *trnL-trnF* was 1.44651, with significant difference at the level of P <0.05, Fu and Li’s F* test statistic was 1.89841, showing a significant difference at the level of P <0.02, and the Tajima’s D was 1.90978, not significant at the level of 0.10> P> 0.05, suggesting its inconformity with the neutral evolution model; the Fu and Li’s D* and F* test statistics for the *ycf1-rps15* were -0.02679 and -0.19784 respectively, and the Tajima’s D was -0.41630, not significant at the level of P> 0.10, in line with the neutral evolution model; the Fu and Li’s D* and F* test statistics of the combined sequence were 0.34297 and 0.08141respectively, and the Tajima’s D was -0.41550, insignificant at the level of P> 0.10, and conforms with the neutral evolution model (Table 4).

**Table 4:** Nucleotide diversity of the intergenic spacers of the cultivated *S*. *miltiorrhiza* populations.

Marker	Hap	Hd	π	Vh	Sh	Fu and Li’s D*	Fu and Li’s F*	Tajima’s D
*ETS*	16	0.64	0.11217	0.00795	0.089	0.22815	-0.37550	-1.27644
*psbA-trnH*	11	0.601	0.08478	0.00763	0.087	0.19802	0.53273	0.90040
*trnL-trnF*	4	0.619	0.03260	0.00326	0.057	1.44651	1.89841	1.90978
*ycf1-rps15*	13	0.795	0.06830	0.00205	0.045	-0.02679	-0.19784	-0.41630
Combined seq.	26	0.950	0.07870	0.0005	0.023	0.34297	0.08141	-0.41550

The results suggested that the three intergenic spacers, *ETS*, *psbA-trnH*, *ycf1-rps15*, and the combined sequence of the cultivated *S*. *miltiorrhiza* populations conform with the neutral evolution at the species level, while the *trnL-trnF* does not.

### Evolutionary relationship of the intergenic spacers

**Fig 2:**
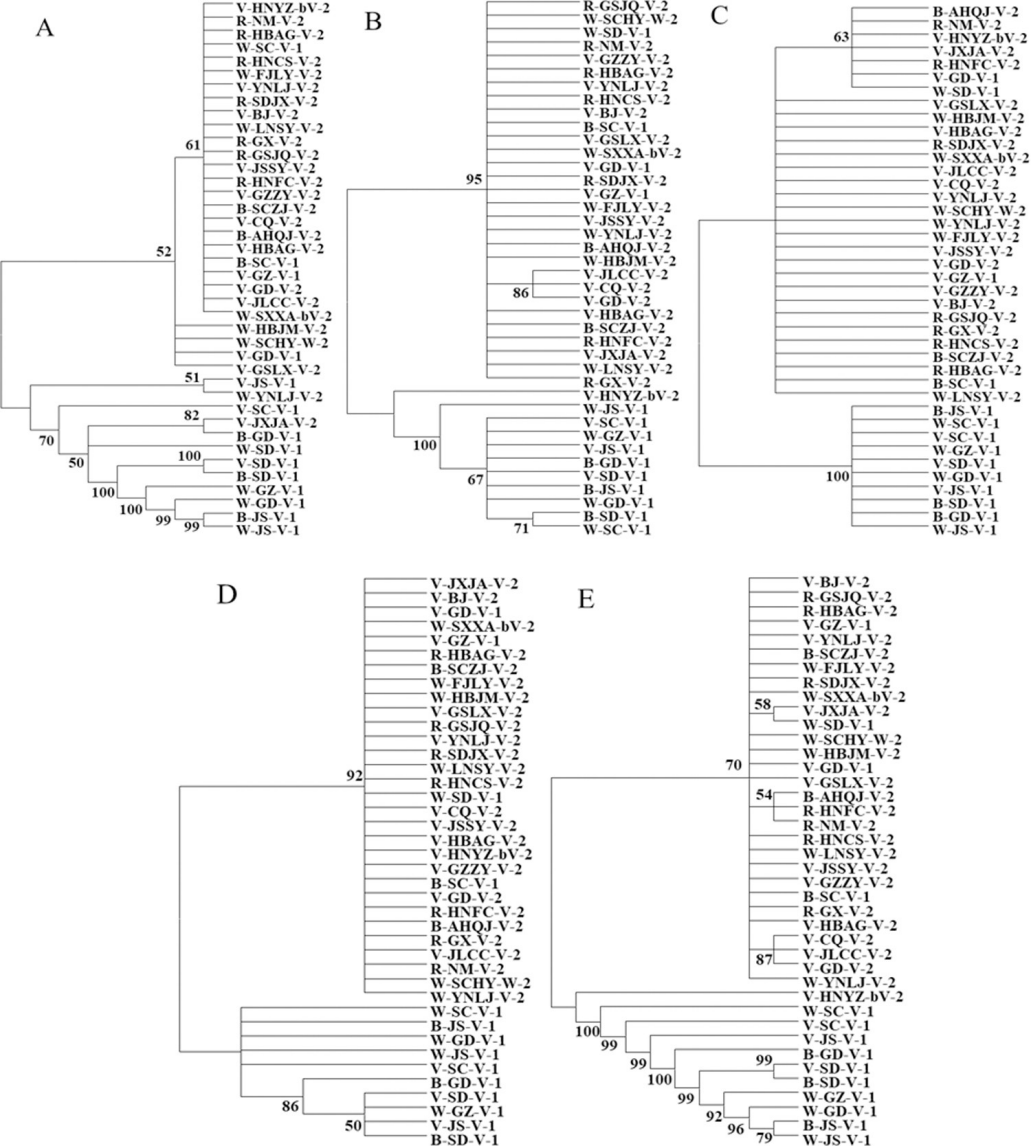
Phylogenetic trees based on the individual and combined intergenic spacers of the cultivated *S*. *miltiorrhiza* populations. A: *ETS;* B: *psbA-trnH*; C: *trnL-trnF*; D: *ycf1-rps15;* E: Combined sequence.

Unrooted phylogenetic trees with a cut-off value of 50% for consensus based on the four intergenic spacers (*ETS*, *psbA-trnH*, *trnL-trnF*, and *ycf1-rps15*) of the 40 cultivated *Danshen* populations were constructed. The phylogenetic trees based on *ETS* and *psbA-trnH* of *S*. *miltiorrhiza* were similarly shown a two-clade structure: for *ETS*, 28 populations clustered as one, and the remaining 12 populations clustered in the other clade (Fig 2A), and for *psbA-trnH*, 29 populations clustered in one clade, and the remaining 11 in the other (Fig 2B). The phylogenetic trees based on the *trnL-trnF* and *ycf1-rps15* of *S*. *miltiorrhiza* showed a very similar structure in that the same 30 populations clustered in one clade, and the remaining 10 clustered in the other (Fig 2C and 2D). The phylogenetic tree based on the combined sequence of *S*. *miltiorrhiza* showed that 40 populations clustered in 2 clades, among which 29 populations are clustered in one clade, and the remaining 11 populations clustered in the other (Fig 2E), very similar to *psbA-trnH* (Fig 2B). The four intergenic spacers and the combined sequence of the 40 cultivated *S*. *miltiorrhiza* populations all showed a similar two-clade topological structure in the phylogenetic trees with only slight differences.

## Discussion and conclusion

For phylogenetic and genetic diversity studies, the key is the selection of suitable genetic markers [37]. It has been suggested that DNA barcodes are used for species identification by sequencing the standard regions of DNA [38]. Although the ribosomal DNA contains very rich genetic information and undergoes a high rate of evolution, it is often prone to recombination and heterozygous due to the biparental inheritance. It is difficult to find single- or low-copy nuclear genes with sufficient variation [39]. Thus it is often confronted with the problem of orthology or paralogy in pedigree geography studies. In contrast, chloroplast DNA is generally maternally inherited in most angiosperms [40] and its non-coding regions are subject to low selective pressures with no gene recombination, which can unambiguously reflect the pedigree history, and has gradually been widely used in studies of plant genetic structure evaluation, population genetic diversity, phylogeny and pedigree geography [41]. The nuclear ribosomal *ETS* evolve rapidly and have high polymorphism and variability, and play important roles in the studies of genetic variation, classification and phylogeny. However, it is difficult to find suitable universal primers to amplify the entire region of *ETS*. So there are relatively few related reports in systematic studies [42].

In this study, the four intergenic spacers of *ETS*, *psbA-trnH*, *trnL-trnF* and *ycf1-rps15* of the 40 cultivated *S*. *miltiorrhiza* populations in China were successfully amplified by PCR. Based on the matrix length, the number of parsimony informative sites accounted for 205, 71, 24, and 88, respectively (Table 3). The results indicate that, among single markers, the *ETS* of the cultivated *S*. *miltiorrhiza* populations were the highest number of parsimony information sites. The PIS rates (Table 3) were generally consistent with the study of the phylogenetic relationship and haplosystem of Lamiaceae plants [26], which showed parsimony information rates of 39.8, 23.95, and16.67%, respectively, for the *ETS*, *ycf1-rps15*, and *trnL-trnF*, markers. The lower PIS of the chloroplast *IGS* than that of *ETS* demonstrated both in our research and in haplosystem of Lamiaceae plants [26] indicate that *ETS* within the nuclear genome are more varied, more informative, and more discriminating than those *IGS* in the chloroplast genome (*psbA-trnH*, *trnL-trnF*, and *ycf1-rps15*). However, the number of parsimony informative sites of the combined sequence was even higher (392), reaching a discrimination rate of 52.5%.

It is generally accepted that for most of the common species, the more widely distributed, the higher its genetic diversity, while the genetic diversity of endemic, rare or narrowly distributed species is low, and the low genetic diversity is an important factor leading to species endangerment or even extinction [43]. In this study, the genetic diversity analysis of 40 cultivated populations of *S*. *miltiorrhiza* showed that the Hd of *ETS*, *psbA-trnH*, *trnL-trnF* and *ycf1-rps15* sequences were all greater than 0.5, indicating that *S*. *miltiorrhiza* has very rich genetic diversity at the species level, which is consistent with the result based on *EST-SSR* markers for *S*. *miltiorrhiza* [44]. The Hd of the combined sequences is 0.950, much higher than that of the four individual intergenic spacers, suggesting the better reflection of genetic diversity of the cultivated *S*. *miltiorrhiza* populations by multi-sequence combination, and could be important in evaluating its genetic variation and adaptability. The best-fit nucleotide substitution models for *ETS*, *psbA-trnH*, *trnL-trnF*, *ycf1-rps15* and the combined sequence are HKY+I, T92, T92, T92+G and T92+G respectively. The lower conversion versus transversion frequency of the joint sequences is consistent with all the three *IGS*s from the chloroplast genome. The relative high nucleotide substitution rates found in this research might be the reason for the high genetic variation among the cultivated *S*. *miltiorrhiza* populations, as suggested [45]. Results from Fu and Li’s D* and F* test statistics, together with the Tajima’s D neutral test show that the *ETS*, *psbA-trnH* and *ycf1-rps15* all conform with the neutral evolution model, while *trnL-trnF* does not. The combined sequence is not significant at the level of P>0.10, and conforms with the neutral evolution model. It is suggested that the cultivated *S*. *miltiorrhiza* populations has not recently experienced expansion events.

It was also found, in this research, that the phylogenetic trees based on both the individual and the combined sequences of the four intergenic spacers of the 40 cultivated populations of *S*. *miltiorrhiza* exhibited topologically very similar two-clade structures. For single sequences, *ETS* has the highest identification rate of 35.0%, and the *trnL-trnF* sequence has the lowest identification rate of 10.0%. The combined DNA marker has an identification rate as high as 52.5%. But still, they could not discriminate all the tested populations. Further endeavor to explore more and effective DNA markers for the identification of more reliable composite molecular markers is needed so that convenient, fast, efficient and standard identification at the gene level could be established.

## Supporting information

S1 Table GenBank accessions of the four intergenic spacers of the 40 cultivated *S*. *miltiorrhiza* populations. (DOCX)
