# Supplementary material for: Genetic diversity of the cultivated Salvia miltiorrhiza populations revealed by four intergenic spacers
Source: PLoS One. 2022 Apr 6;17(4):e0266536. doi: 10.1371/journal.pone.0266536 (PMC8985983; doi:10.1371/journal.pone.0266536)

Table S1 *GenBank accessions of the four intergenic spacers of the 40 cultivated S. miltiorrhiza populations*

Population/

GenBank Accession

psbA-trnH trnL-trnF

Population/

Voucher

GenBank Accession

psbA-trnH trnL-trnF

Voucher

ETS

ycf1-rps15

ETS

ycf1–rps15

V-HBAG-V-2

V-CQ-V-2

MW699307

MW699308

MZ329816

MZ329817

MZ318203

MZ318204

MZ292608

MZ292609

B-SCZJ-V-2

B-SD-V-1

MW699327

MW699328

MZ329836

MZ329837

MZ318223

MZ318224

MZ292628

MZ292629

V-JXJA-V-2

V-JLCC-V-2

V-JSSY-V-2

V-GZ-V-1

MW699309

MW699310

MW699311

MW699312

MW699313

MW699314

MW699315

MW699316

MW699317

MW699318

MW699319

MW699320

MW699321

MW699322

MW699323

MW699324

MW699325

MW699326

MZ329818

MZ329819

MZ329820

MZ329821

MZ329822

MZ329823

MZ329824

MZ329825

MZ329826

MZ329827

MZ329828

MZ329829

MZ329830

MZ329831

MZ329832

MZ329833

MZ329834

MZ329835

MZ318205

MZ318206

MZ318207

MZ318208

MZ318209

MZ318210

MZ318211

MZ318212

MZ318213

MZ318214

MZ318215

MZ318216

MZ318217

MZ318218

MZ318219

MZ318220

MZ318221

MZ318222

MZ292610

MZ292611

MZ292612

MZ292613

MZ292614

MZ292615

MZ292616

MZ292617

MZ292618

MZ292619

MZ292620

MZ292621

MZ292622

MZ292623

MZ292624

MZ292625

MZ292626

MZ292627

R-HBAG-V-2

R-HNFC-V-2

R-SDJX-V-2

R-GX-V-2

MW699329

MW699330

MW699331

MW699332

MW699333

MW699334

MW699335

MW699336

MW699337

MW699338

MW699339

MW699340

MW699341

MW699342

MW699343

MW699344

MW699345

MW699346

MZ329838

MZ329839

MZ329840

MZ329841

MZ329842

MZ329843

MZ329844

MZ329845

MZ329846

MZ329847

MZ329848

MZ329849

MZ329850

MZ329851

MZ329852

MZ329853

MZ329854

MZ329855

MZ318225

MZ318226

MZ318227

MZ318228

MZ318229

MZ318230

MZ318231

MZ318232

MZ318233

MZ318234

MZ318235

MZ318236

MZ318237

MZ318238

MZ318239

MZ318240

MZ318241

MZ318242

MZ292630

MZ292631

MZ292632

MZ292633

MZ292634

MZ292635

MZ292636

MZ292637

MZ292638

MZ292639

MZ292640

MZ292641

MZ292642

MZ292643

MZ292644

MZ292645

MZ292646

MZ292647

V-GD-V-1

V-GD-V-2

V-GSLX-V-2

V-BJ-V-2

R-NM-V-2

R-HNCS-V-2

R-GSJQ-V-2

W-SCHY-W-1

W-SXXA-bV-2

W-LNSY-V-2

W-FJLY-V-2

W-GZ-V-1

V-YNLJ-V-2

V-GZZY-V-2

V-SC-V-1

V-SD-V-1

V-JS-V-1

W-SD-V-1

V-HNYZ-bV-2

B-SC-V-1

W-JS-V-1

W-SC-V-1

B-AHQJ-V-2

B-GD-V-1

B-JS-V-1

W-GD-V-1

W-HBJM-V-2

W-YNLJ-V-2

1


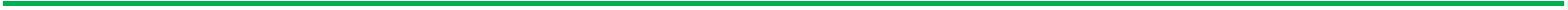

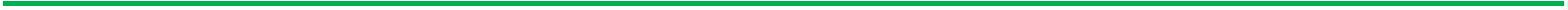

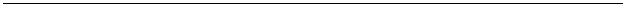

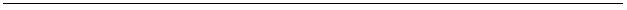

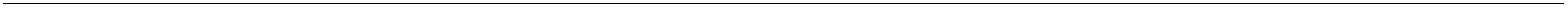

Supplement: S1 Table — (DOCX) [file pone.0266536.s001.docx]
